# High-Density Lipoprotein (HDL) Counter-Regulates Serum Amyloid A (SAA)-Induced sPLA_2_-IIE and sPLA_2_-V Expression in Macrophages

**DOI:** 10.1371/journal.pone.0167468

**Published:** 2016-11-29

**Authors:** Shu Zhu, Yongjun Wang, Weiqiang Chen, Wei Li, Angelina Wang, Sarabeth Wong, Guoqiang Bao, Jianhua Li, Huan Yang, Kevin J. Tracey, John D’Angelo, Haichao Wang

**Affiliations:** 1 Department of Emergency Medicine, North Shore University Hospital, Manhasset, New York, United States of America; 2 The Feinstein Institute for Medical Research, Manhasset, New York, United States of America; 3 Department of General Surgery, Tangdu Hospital, The 4^th^ Military Medical University, Xi'an, Shaanxi, China; University of North Dakota, UNITED STATES

## Abstract

Human serum amyloid A (SAA) has been demonstrated as a chemoattractant and proinflammatory mediator of lethal systemic inflammatory diseases. In the circulation, it can be sequestered by a high-density lipoprotein, HDL, which carries cholesterol, triglycerides, phospholipids and apolipoproteins (Apo-AI). The capture of SAA by HDL results in the displacement of Apo-AI, and the consequent inhibition of SAA’s chemoattractant activities. It was previously unknown whether HDL similarly inhibits SAA-induced sPLA_2_ expression, as well as the resultant HMGB1 release, nitric oxide (NO) production and autophagy activation. Here we provided compelling evidence that human SAA effectively upregulated the expression and secretion of both sPLA_2_-IIE and sPLA_2_-V in murine macrophages, which were attenuated by HDL in a dose-dependent fashion. Similarly, HDL dose-dependently suppressed SAA-induced HMGB1 release, NO production, and autophagy activation. In both RAW 264.7 cells and primary macrophages, HDL inhibited SAA-induced secretion of several cytokines (e.g., IL-6) and chemokines (e.g., MCP-1 and RANTES) that were likely dependent on functional TLR4 signaling. Collectively, these findings suggest that HDL counter-regulates SAA-induced upregulation and secretion of sPLA_2_-IIE/V in addition to other TLR4-dependent cytokines and chemokines in macrophage cultures.

## Introduction

Harboring various fatty acid side chains and phospholipid head groups [e.g., phosphatidylcholine (PC), phosphatidylserine (PS), or phosphatidyl ethanolamine (PE)], the heterogeneous phospholipids serve as the major components of cytoplasmic membranes and lipoprotein particles. The A_2_ group of phospholipases (PLA_2_s) hydrolyzes the fatty acid at the sn-2 position of the glycerol backbone of the phospholipids, releasing lysophospholipid as well as free fatty acids such as arachidonic acid (AA)–a substrate for other signaling lipids including prostaglandin E_2_ (PGE_2_), leukotrienes, and eicosanoids. Based on their molecular weight, cellular localization and dependence on calcium, PLA_2_s are further subdivided into: 1) Ca^2+^-dependent cytosolic enzymes (cPLA_2_s); 2) the low-molecular-weight and Ca^2+^-dependent secretory PLA_2_s (sPLA_2_); 3) Ca^2+^-independent enzymes (iPLA_2_s); 4) lipoprotein-associated PLA_2_ (Lp-PLA_2_ or sPLA_2_-VII); 5) lysosomal enzymes (LPLA_2_); and 6) adipose-specific enzymes (AdPLA_2_s) [1]. In general, different sPLA_2_s participate in diverse processes ranging from generating lipid metabolites, promoting membrane remodeling, and modifying extracellular lipid components (e.g., lipoproteins), to degrading phospholipids in invading pathogens and ingesting dietary components. For instance, the mammalian sPLA_2_ family contains 10 catalytically active isoforms (IB, IIA, IIC, IID, IIE, IIF, III, V, X, and XIIA) [1], which predominantly hydrolyze phospholipids in the extracellular environment.

During inflammation, innate immune cells (macrophages and monocytes) sequentially release early cytokines (e.g., TNF, IL-1, and IFN-γ) [2] and late proinflammatory mediators such as sPLA_2_ [1], nitric oxide (NO) [3] and HMGB1 [4]. As a cascade response, early cytokines can further stimulate innate immune cells to release sPLA_2_ [5], which potentiates the subsequent release of NO [6] and HMGB1 [7]. Additionally, early cytokines also alter the expression of liver-derived acute phase proteins, which then participate in the regulation of late proinflammatory mediators.

For instance, TNF, IL-1β and IFN-γ induce the expression of serum amyloid A (SAAs) in both hepatocytes [8] and innate immune cells (e.g., macrophages/monocytes) [9]. Overall, the human SAA family is comprised of multiple members including the most abundant SAA1, and other less prominent isoforms such as SAA, SAA2α, SAA2β, and SAA3. Following endotoxemia, circulating SAA levels are dramatically elevated (up to 1000-fold) within 16–24 h as a result of the de novo expression of early cytokine inducers and subsequent synthesis of SAAs [10,11]. Upon secretion, extracellular SAA signals via a family of receptors including the receptor for advanced glycation end products (RAGE) [12], TLR2 [13,14], TLR4 [15], P2X7 receptor [16], and pertussis toxin-sensitive receptors [e.g., formyl peptide receptor 2 (FPR2)] [17], thereby inducing various cytokines and chemokines (e.g., TNF, IL-1β, IL-6, G-CSF, IL-8, MCP-1, MIP-1α, and MIP-3α) [18,19]. It also serves as a chemoattractant for inflammatory cells such as macrophages/monocytes [17,20,21] and T cells [22]. Interestingly, SAA can stimulate smooth muscle cells to release sPLA_2_-IIA [23], and induce human THP-1 monocytes to express lipoprotein-associated PLA_2_ (Lp-PLA_2_ or sPLA_2_-VII) [24].

SAA contains an N-terminal α-helical domain (amino acid 1–28) capable of binding high-density lipoproteins (HDL) [25,26], the smallest lipoproteins that carry cholesterol, triglycerides, and phospholipids within the water-based blood stream. The capture of SAA by HDL results in the displacement of apolipoproteins (Apo-AI) and formation of larger HDL particles (up to 200 kDa) [27,28]. At physiologically relevant concentrations (>100 μg/ml), HDL almost completely blocks the chemoattractant activities of SAA [20], suggesting HDL as a natural inhibitor of SAA in the circulation. Although we recently demonstrated that SAA stimulates macrophages to release HMGB1 (29), it was previously unknown whether SAA also upregulates sPLA_2_ secretion, an essential prerequisite for NO production [6] and HMGB1 release [7]. In this study, we sought to examine whether human SAA upregulated the expression and secretion of sPLA_2_s in macrophage cultures. Furthermore, we determined whether HDL suppressed SAA-induced sPLA_2_ expression, HMGB1 release, NO production, autophagy activation, or secretion of other cytokines and chemokines.

## Materials and Methods

### Materials

Dulbecco's Modified Eagle's Medium (DMEM, Cat. No. 11995–065), penicillin/streptomycin (Cat. No. 15140–122), and fetal bovine serum (FBS, Cat. No. 26140079) were obtained from Invitrogen (Grand Island, New York). OPTI-MEM I reduced serum medium (Cat. No. 31985062), the Trizol reagent (Cat. No. 15596–026), and the RevertAid^™^ First Strand cDNA Synthesis Kit (Cat. No. K1621) were obtained from Thermo Fisher Scientific (Springfield, New Jersey). The RT^2^ SYBR Green ROX qPCR Mastermix (Cat. No. 330521) was obtained from the Qiagen (Valencia, CA). Purified high-density lipoprotein (HDL, Cat. No. L8039, >95% purity) and anti-β-actin antibody (Cat. No. A1978) were obtained from Sigma-Aldrich (St. Louis, MO). Recombinant human SAA (also called Apo-SAA, Cat. No. 300–13) was obtained from PeproTech (Rocky Hill, NJ). The apo-SAA is almost identical to human Apo-SAA1α, except for the presence of an N-terminal methionine, the substitution of asparagine for aspartic acid at position 60, and arginine for histidine at position 71, the latter two substituted residues are present in Apo-SAA2β. HRP conjugated donkey anti-rabbit IgG was from GE Healthcare (Cat. No. NA934). HMGB1-specific polyclonal antibodies were generated in rabbits as previously described [4]. Two lines of sPLA_2_-reacting antibodies (Cat. No. ab23709 and Cat. No. ab139692) were obtained from Abcam (Cambridge, MA). LC3 mouse monoclonal antibody (Cat. No. SC-16755) was obtained from Santa Cruz Biotechnology. TLR2, TLR4 and RAGE KO, and TLR2/RAGE and TLR4/RAGE-double KO mice on a C57BL/6 genetic background were maintained at The Feinstein Institute for Medical Research as previously described [29]. Because the KO mice were derived from C57BL/6 mice, small colonies of wild-type C57BL/6 (Jackson Laboratory) were maintained under the same conditions.

### Cell culture

Primary peritoneal macrophages were isolated from Balb/C mice (Taconic, male, 7–8 weeks, 20–25 g), wild-type C57BL/6, or various SAA receptor knockout mice (male, 7–8 weeks, 20–25 g) at 2–3 days after intraperitoneal injection of 2 ml thioglycollate broth (4%) as previously described [30,31]. Briefly, Balb/C or C57BL/6 mice were sacrificed by CO_2_ asphyxiation, and the abdomen region was cleaned with 70% ethanol before making a small excision of the abdominal skin to expose the abdominal wall, and to insert a catheter into viscera-free pocket in order to wash out peritoneal macrophages with 7.0 ml of 11.6% sucrose solution. This study was approved by the Institutional Animal Care and Use Committee (IACUC protocol #: 2008–033; Approval date: September 28^th^, 2012), and performed in accordance with the guidelines for the care and use of laboratory animals at the Feinstein Institute for Medical Research, Manhasset, New York. Murine macrophage-like RAW 264.7 cells were obtained from the American Type Culture Collection (ATCC, Rockville, MD). RAW 264.7 macrophages and primary macrophages were cultured in DMEM supplemented with 1% penicillin/streptomycin and 10% FBS. When reaching 70–80% confluence, adherent macrophages were gently washed with, and cultured in, OPTI-MEM I before stimulating with human SAA, in the absence or presence of HDL for 16 h. Subsequently, the cell-conditioned culture media were analyzed respectively for the levels of sPLA_2_, HMGB1, nitric oxide (NO), and other cytokines by Western blotting analysis, the Griess Reaction, and Cytokine Antibodies Arrays as previously described [32,33].

### Western blotting

The levels of sPLA_2_ and HMGB1 in the culture medium were determined by Western blotting analysis as previously described [4,34]. Briefly, an equal volume of culture medium (conditioned by identical macrophage cell numbers) was harvested, and protein content was concentrated by ultrafiltration (with a molecular weight cutoff of 3.0 kDa), and then normalized to the same volume with a sample buffer. Proteins in equal sample volume were resolved on sodium dodecyl sulfate (SDS)-polyacrylamide gels, and then transferred to polyvinylidene difluoride (PVDF) membranes. After blocking with 5% non-fat milk, the membrane was incubated with respective antibodies (anti-sPLA_2_, 1:500; anti-HMGB1, 1:1000) overnight. Subsequently, the membrane was incubated with the appropriate secondary antibodies, and the immunoreactive bands were visualized by chemiluminescence technique.

The basic principle of autophagy assay was to measure the biochemical conversion of the endogenous LC3-I to phosphatidylethanolamine (PE)-conjugated LC3-II by Western blotting analysis [35]. Although the actual molecular weight of PE-conjugated-LC3-II (16 kDa) is larger than that of LC3-I (14 kDa), LC3-II migrates faster than LC3-I in SDS-PAGE because of its higher hydrophobicity. The ratio between the 16-kD lipidated LC3-II and a house-keeping protein, β-actin, was determined by Western blotting analysis as previously described [33].

### Real-time RT-PCR

Total RNA was isolated from murine macrophages using the Trizol reagent kit as per the manufacturer’s instructions, and reversely transcribed into the first-strand cDNA using the RevertAid^™^ First Strand cDNA Synthesis Kit. Following reverse transcription, a panel of established primers for murine pla2g2a (Qiagen, QT00109977), pla2g2d (QT00120638), pla2g2e (QT01049125), pla2g2f (QT00173838), pla2g5 (QT00197806), and glyceraldehyde 3-phosphate dehydrogenase gene (Gapdh; QT01658692) was used to quantify the mRNA expression levels of respective genes using a ABI 7900HT Fast Real-time PCR system (Applied Biosystems, Foster City, CA). Amplification was performed using the RT² SYBR Green ROX qPCR Mastermix under the following conditions: 95°C 10’; followed by 40 cycles of 95°C for 15” and 60°C for 1’. Immediately following the amplification step, a single cycle of the dissociation (melting) curve program was run at 95°C for 15”, then at 60°C for 15”, and last at 95°C for 15”. This cycle was followed by a melting curve analysis, baseline and cycle threshold values (Ct values) were automatically determined using the ABI 7900HT software. The relative sPLA_2_ mRNA expression was calculated using the following formula: ΔΔC expression = 2^–Δ ΔCt^, where ΔΔCt = 0394Ct (treated group)– ΔCt (control group), ΔCt = Ct (target gene)–Ct (GAPDH), and Ct = cycle at which the threshold was reached. The relative abundance of sPLA_2_ mRNA expression in control group was set as an arbitrary unit of 1, and the gene expression in treated groups was presented as folds of controls after normalization to GAPDH.

### Nitric oxide (NO) assay

The levels of NO in the culture medium were determined indirectly by measuring the NO^2−^ production with a colorimetric assay based on the Griess reaction [30,36]. NO^2−^ concentrations were determined with reference to a standard curve generated with sodium nitrite at various dilutions.

### LC3 aggregation

Another basic principle of autophagy assay was to measure the transfer of a soluble and membrane-impermeant LC3 protein from cytosol to autophagic vesicles (autophagosomes) [35]. To visualize LC3-containing cytoplasmic vesicles, GFP-LC3-transfected RAW 264.7 cells were stimulated with SAA (2.0 μg/ml) in the absence or presence of HDL (100 μg/ml) for 16 h, and examined for the formation of GFP-LC3 punctate structures under a fluorescence microscope as previously described [33].

### Cytokine antibody array

Murine Cytokine Antibody Arrays (Cat. No. M0308003, RayBiotech Inc., Norcross, GA, USA), which respectively detect 62 cytokines on one membrane, were used to determine cytokine levels in macrophage-conditioned culture medium as previously described [30,36]. Briefly, the membranes were sequentially incubated with equal volumes cell-conditioned culture medium (200 μl), primary biotin-conjugated antibodies, and horseradish peroxidase—conjugated streptavidin. After exposing to X-ray film, the relative signal intensity was determined using the Scion Image software.

### Statistical analysis

Data are expressed as mean ± SD of three independent experiments (n = 3). One-way analyses of variance (ANOVA) followed by the Tukey’s test for multiple comparisons were used to compare between different groups. A *P* value less than 0.05 was considered statistically significant.

## Results

### HDL inhibited SAA-induced release of sPLA_2_ in macrophage cultures

**Fig 1:**
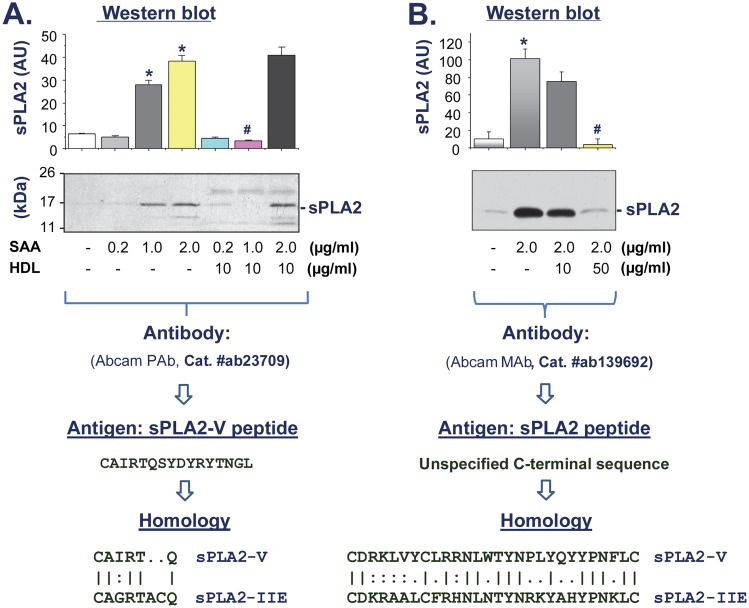
HDL attenuated SAA-induced sPLA_2_ secretion in murine macrophage-like RAW 264.7 cells. RAW 264.7 cells were stimulated with SAA in the absence or presence of HDL at indicated concentrations for 16 h, and the extracellular levels of sPLA_2_ in the macrophage-conditioned medium were determined by Western blotting using two different antibodies raised against a sPLA_2_-V peptide (**Panel A**) or a unspecified peptide in the homologous C-terminus of sPLA_2_s (**Panel B**). The cross-reactivity of these Abcam antibodies was illustrated. Sample loading was normalized by equal volume of culture medium conditioned by equal number of cells. Bar graph is a summary of three experiments. *, *P* < 0.05 versus “-SAA” controls; #, *P* < 0.05 versus “+ SAA” control.

It has been shown that SAA can stimulate smooth muscle cells to release sPLA_2_ [23], and human THP-1 monocytes to express lipoprotein-associated PLA_2_ (Lp-PLA_2_ or sPLA_2_-VII) [24]. To assess whether SAA induces sPLA_2_ in innate immune cells, murine macrophage-like RAW 264.7 cells were stimulated with SAA at various concentrations (0.2, 1.0, 2.0 μg/ml) for 16 h, and the extracellular levels of sPLA_2_ in the macrophage-conditioned medium were determined by Western blotting using two different antibodies: rabbit polyclonal antibodies against a sPLA_2_-V peptide (Fig 1A), and rabbit monoclonal antibody against a peptide in the homologous C-terminus of sPLA_2_s (Fig 1B). At pathophysiologically relevant concentrations, SAA stimulated the secretion of sPLA_2_ in a dose-dependent fashion (Fig 1A), suggesting SAA as a positive regulator of sPLA_2_ in innate immune cells.

It has been shown that HDL, at physiologically relevant concentrations (>100 μg/ml), can capture SAA [27,28] and attenuate its chemoattractant activities [20]. To test whether HDL similarly affects SAA-induced sPLA_2_ secretion, macrophages were stimulated with SAA in the presence of HDL at various concentrations. As indicated in Fig 1, HDL effectively inhibited SAA-induced sPLA_2_ secretion in a dose-dependent manner, with an almost complete abrogation when HDL was given at a higher concentration (Fig 1A and 1B).

**Fig 2:**
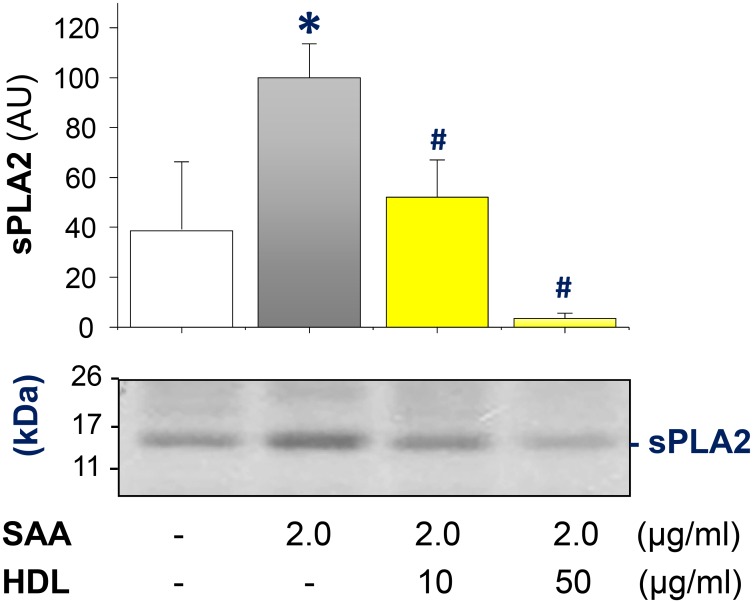
HDL inhibited SAA-induced sPLA_2_ secretion in thioglycollate-elicited primary peritoneal macrophages. Thioglycollate-elicited primary macrophages were isolated from Balb/C mice, and stimulated by SAA (2.0 μg/ml) in the absence or presence of HDL for 16 h, and extracellular levels of sPLA_2_ were determined by Western blotting using antibody raised against a unspecified conserved peptide in the C-terminus of sPLA_2_s (Abcam, Cat. # ab139692). Bar graph is a summary of three experiments. *, *P* < 0.05 versus “-SAA” controls; #, *P* < 0.05 versus “+ SAA” control.

To assess its physiological relevance, thioglycollate-elicited peritoneal macrophages were isolated from Balb/C mice, and the experiments were repeated under similar conditions. Consistent with a previous notion that elicited peritoneal macrophages released sPLA_2_s [37], we found that thioglycollate-elicited primary murine macrophages also “constitutively” secreted sPLA_2_ even in the absence of SAA stimulation (Fig 2). However, SAA could further elevate the extracellular sPLA_2_ levels up to 1.5-fold. Similarly, HDL dose-dependently and significantly prevented SAA-induced sPLA_2_ secretion. At higher concentrations, HDL even further reduced extracellular sPLA_2_ to below basal levels (Fig 2). It remains elusive whether HDL directly binds and removes sPLA_2_ or trivial contaminating endotoxins, that could also induce sPLA_2_ secretion, from the cell-conditioned culture medium through receptor-mediated endocytosis. Thus, HDL can counter-regulate SAA-induced sPLA_2_ secretion in both RAW 264.7 cells and primary macrophage cultures.

### HDL dose-dependently prevented SAA-induced up-regulation of sPLA_2_-IIE and sPLA_2_-V mRNA

**Fig 3:**
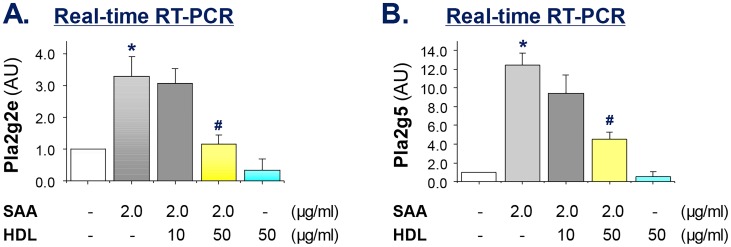
HDL dose-dependently suppressed SAA-induced mRNA up-regulation of pla2g2e and pla2g5 in macrophage cultures. Murine RAW 264.7 cells were stimulated with SAA in the absence or presence of HDL at indicated concentrations for 16 h, and the cellular levels of pla2g2e and pla2g5 mRNA were determined by real-time RT-PCR, and expressed as mean ± SD of Gapdh mRNA levels (in arbitrary units, AU) of three independent experiments. *, *P* < 0.05 versus “-SAA” controls; #, *P* < 0.05 versus “+ SAA” control.

To elucidate the molecular mechanism by which HDL inhibited SAA-induced sPLA_2_ secretion, we measured their mRNA expression levels by real-time RT-PCR using commercially available primers specific for several sPLA_2_s. Surprisingly, SAA did not significantly induce the gene expression of sPLA_2_-IIA, sPLA_2_-IID and sPLA_2_-IIF in murine macrophage cultures (data not shown). In a sharp contrast, SAA reproducibly and significantly increased the mRNA expression levels of sPLA_2_-IIE and sPLA_2_-V by 3.5- (Fig 3A) and 12-fold (Fig 3B), respectively. By itself, HDL did not significantly alter the basal mRNA expression of sPLA_2_, but dose-dependently inhibited SAA-induced up-regulation of sPLA_2_-IIE (Fig 3A) and sPLA_2_-V mRNA (Fig 3B). Taken together, these findings suggest that SAA effectively up-regulated sPLA_2_-IIE and sPLA_2_-V in murine macrophage cultures, which can be counter-regulated by HDL, confirming HDL as an endogenous SAA antagonist.

### HDL dose-dependently prevented SAA-induced HMGB1 release

**Fig 4:**
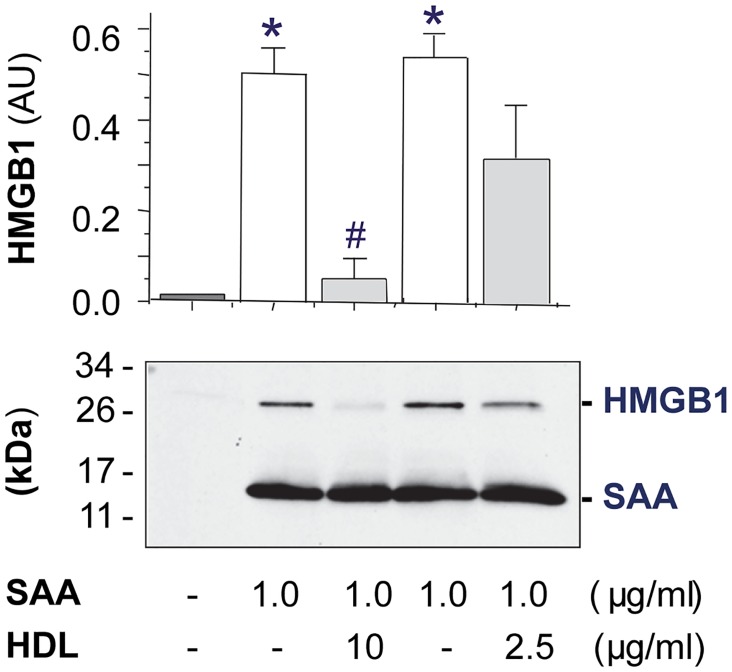
HDL dose-dependently prevented SAA-induced HMGB1 release. Murine macrophages were stimulated with SAA in the absence or presence of HDL for 16 h, and extracellular HMGB1 levels were determined by Western blotting analysis. Shown in the bar graph is a summary of three independent experiments. *, *P* < 0.05 versus “-SAA” controls; #, *P* < 0.05 versus “+ SAA” control.

It has been suggested that the bacterial endotoxin-induced HMGB1 release is precipitated by the upregulated secretion of sPLA_2_ in innate immune cells [7], suggesting that agents capable of inhibiting sPLA_2_ secretion may also suppress HMGB1 release. To test this possibility, we stimulated murine macrophages with SAA in the absence or presence of HDL, and measured the levels of HMGB1 release by Western blotting analysis. Consistent with our recent report [29], SAA effectively induced HMGB1 release in murine macrophage cultures (Fig 4). As predicted, HDL effectively prevented SAA-induced HMGB1 release in a dose-dependent fashion (Fig 4), supporting a previous notion that active HMGB1 release might be dependent on the prerequisite sPLA_2_ secretion in innate immune cells.

### HDL inhibited SAA-induced NO production and autophagy activation

**Fig 5:**
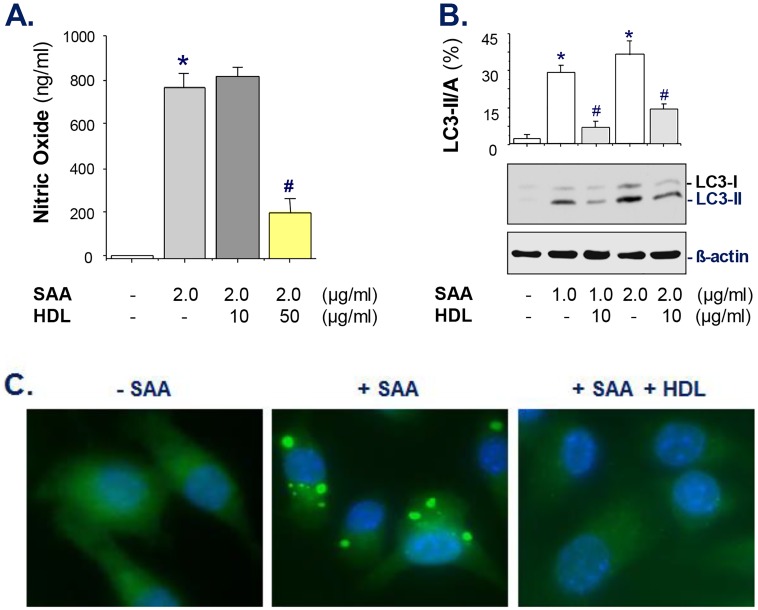
HDL dose-dependently inhibited SAA-induced NO production and autophagy induction. A). HDL inhibited SAA-induced NO production. RAW 264.7 cells were stimulated with SAA in the absence or presence of HDL for 16 h, and extracellular NO levels were determined by Griess reaction. Shown in the bar graph is a summary of three independent experiments. *, *P* < 0.05 versus “-SAA” controls; #, *P* < 0.05 versus “+ SAA” control. B, C). HDL inhibited SAA-induced LC3-II production and aggregation. GFP-LC3-transfected RAW 264.7 cells were stimulated with SAA in the absence or presence of HDL for 16 h, and cellular LC3-II levels were determined by Western blotting (**Panel B**). *, *P* < 0.05 versus “-SAA” controls; #, *P* < 0.05 versus “+ SAA” control. In parallel, the formation of LC3 punctuates were examined under fluorescent microscopy (**Panel C**).

Given the important role of sPLA_2_-II in potentiating the expression of the inducible NO synthase (iNOS) and the production of NO [6], we tested whether HDL similarly inhibited SAA-induced NO production. Although at a relative low concentration (10 μg/ml), HDL did not significantly reduce SAA-induced NO production, it promoted a significant inhibition (>75%) when given at a relative higher concentration (Fig 5A).

Previous studies have suggested NO as a signaling molecule for autophagy induction [38], because an iNOS inhibitor, L-NMMA, effectively inhibited LPS/IFN-γ-induced autophagy. We thus tested whether HDL can inhibit SAA-induced autophagy in macrophage cultures. For the first time, we found that SAA dose-dependently and significantly elevated LC3-II production (Fig 5B), which was significantly inhibited by HDL even when given at a relative lower concentration (10 μg/ml). When given at a higher concentration (100 μg/ml), HDL almost completely abrogated SAA (2.0 μg/ml)-induced LC3-II production (data not shown), suggesting that HDL similarly attenuated SAA-induced autophagy. Indeed, the SAA-induced GFP-LC3 aggregation was almost completely abrogated by HDL when given at a higher concentration (100 μg/ml, Fig 5C), further supporting the possibility that HDL effectively inhibited SAA-induced autophagy in macrophage cultures.

### HDL inhibited SAA-induced secretion of several cytokines and chemokines

**Fig 6:**
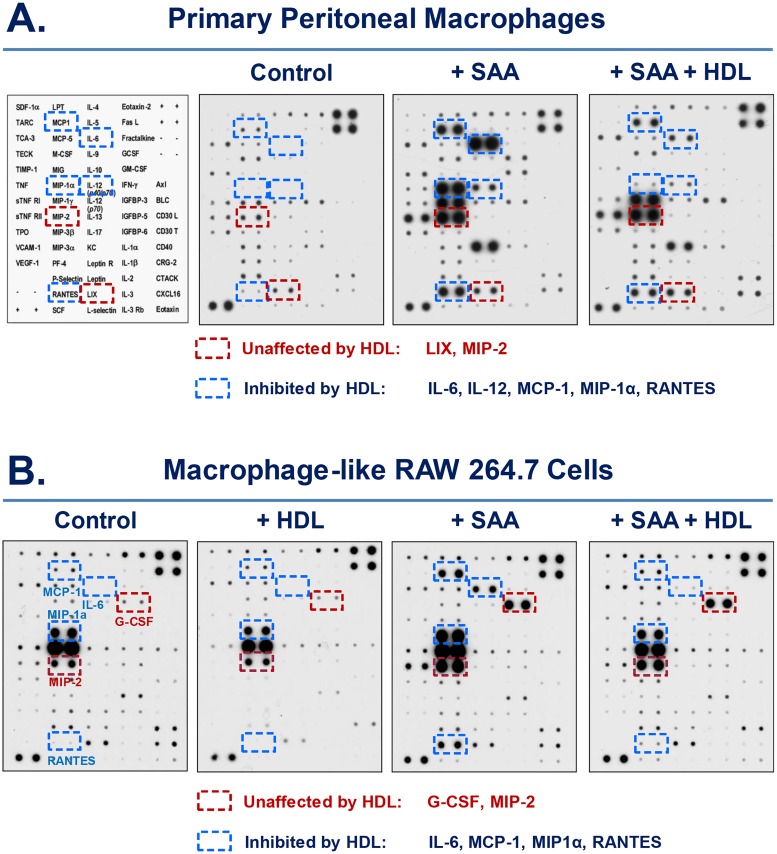
HDL attenuated SAA-induced release of similar cytokines and chemokines in primary macrophages and RAW 264.7 cell line. Thioglycollate-elicited primary macrophages and RAW 264.7 cells were stimulated with recombinant SAA (2.0 μg/ml) in the absence or presence of HDL (100 μg/ml) for 16 h, and the extracellular levels of cytokines and chemokines were determined by Cytokine Antibody Arrays.

To gain a comprehensive understanding of the HDL-mediated counter-regulation of SAA-induced inflammatory response, we examined the effect of HDL on SAA-induced cytokine and chemokine secretion using both primary macrophages and RAW 264.7 cells. As predicted, SAA induced a similar profile of cytokine and chemokine secretion in both types of macrophage cultures (Fig 6A and 6B). Interestingly, HDL conferred a similar inhibition of several cytokines (e.g., IL-6) and chemokines (e.g., MCP-1, MIP-1α, RANTES) in both SAA-stimulated primary macrophages (Fig 6A) and RAW 264.7 cells (Fig 6B). Even when given at a high concentration (100 μg/ml), HDL did not affect the SAA-induced secretion of LIX and MIP-2 in primary macrophages (Fig 6A) or G-CSF and MIP-2 in RAW 264.7 cells (Fig 6B), suggesting that HDL may differentially counter-regulate SAA-induced secretion of various cytokines/chemokines.

### Role of various receptors in SAA-induced cytokines/chemokines

**Fig 7:**
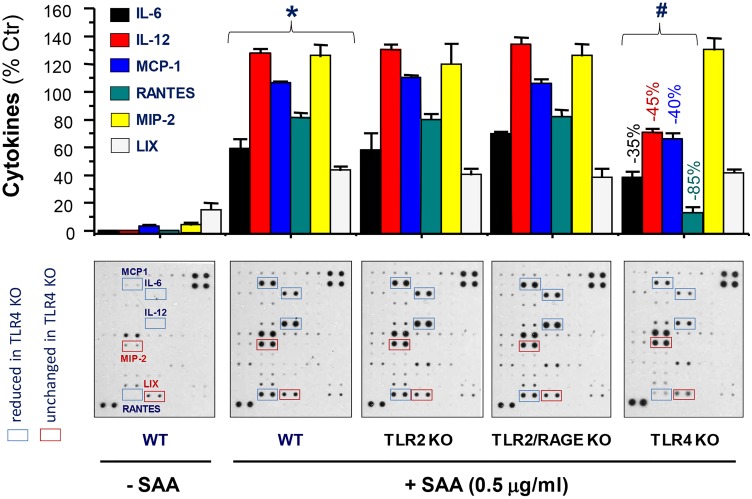
Distinct roles of various receptors in SAA-induced secretion of cytokines/chemokines. Thioglycollate-elicited primary macrophages were isolated from wild-type C56BL/6 or mutant mice respectively deficient in TLR2, TLR4, TLR2/RAGE, or TLR4/RAGE. Following stimulation with SAA (0.5 μg/ml) for 16 h, the extracellular levels of cytokines and chemokines were determined by Cytokine Antibody Arrays. Because the SAA-induced cytokine profiles were superimposable between TLR4- and TLR4/RAGE-deficient macrophages, we only provided results for TLR4 KO macrophages. The experiment was repeated twice. *, *P* < 0.05 versus “-SAA” controls; #, *P* < 0.05 versus “WT + SAA”.

SAAs may employ multiple receptors including RAGE [12], TLR2 [13,14], TLR4 [15], P2X7 receptor [16], and FPR2 [17] to induce various cytokines and chemokines. To identify the receptor(s) responsible for SAA-mediated various cytokines and chemokines, we compared their levels of secretion between primary macrophages of wild-type and mutant mice respectively deficient in TLR2, TLR4, RAGE, and TLR2/RAGE or TLR4/RAGE. The disruption of TLR2, RAGE (data not shown), or TLR2/RAGE did not obviously impair SAA-induced secretion of any cytokines or chemokines (Fig 7). In contrast, the knockout of TLR4 significantly impaired SAA-induced secretion of IL-6, IL-12, MCP-1 and RANTES (Fig 7), but not other chemokines (e.g., MIP-2 and LIX, Fig 7). Likewise, the knockout of both TLR4 and RAGE similarly impaired SAA-induced secretion of IL-6, IL-12, MCP-1 and RANTES, but not other chemokines (e.g., MIP-2 and LIX, data not shown). It suggested that HDL may readily impair SAA-TLR4 signaling to counter-regulate a subset of cytokines and chemokines in macrophage cultures.

## Discussion

In patients with various inflammatory diseases, there are multiple elevated SAA isoforms such as the most abundant SAA1, as well as several scarce variants including SAA, SAA2α, SAA2β, and SAA3 [39]. Despite the high homology between these SAA isomers, their capacities in inducing late proinflammatory mediators (e.g., HMGB1) are dramatically different. We recently discovered that human SAA isomer might be specifically expressed in a subset of septic patients [29], but was capable of inducing HMGB1 release in macrophage and monocyte cultures [29]. In the present study, we provided the first evidence that human SAA also upregulated the expression and secretion of sPLA_2_-IIE and sPLA_2_-V in macrophage cultures.

Among ten members of the sPLA_2_ family, only sPLA_2_-IIE and sPLA_2_-V have been referred to as the “inflammatory” and “metabolic” enzymes for their involvement in regulating innate immune responses and metabolism. Although sPLA_2_-IIE is constitutively expressed in mouse skin hair follicles [40], its expression in adipocytes is upregulated by high-fat diet consumption [41]. Likewise, sPLA_2_-V is also up-regulated in adipocytes of obese mice [41], functioning as another integrated regulator of immune and metabolic responses. Although both enzymes can alter lipoprotein lipid composition, their substrate preference seems to be different. Whereas the sPLA_2_-IIE may hydrolyze PS- or PE-containing lipoproteins; sPLA_2_-V predominantly acts on PC-containing substrates to release lysophospholipid as well as arachidonic acid (AA)–a substrate for other signaling lipids such as prostaglandins, leukotrienes, and eicosanoids.

Although sPLA_2_-IIE was not inducible by LPS in P388D1 and RAW 264.7 macrophage cell lines [42,43], it could be upregulated by IL-1β and HMGB1 in smooth muscle cells [44]. In contrast, sPLA_2_-V could be upregulated by LPS in P388D1 and RAW 264.7 macrophage cell lines [42,43], and even be inducible by anti-inflammatory cytokines (e.g., IL-4) in human macrophages [45]. In the present study, we demonstrated that human SAA effectively upregulated sPLA_2_-IIE and sPLA_2_-V in murine RAW 264.7 cells. Despite the technical difficulty due to paradoxical secretion of sPLA_2_-II from elicited peritoneal macrophages [37], we found that SAA significantly elevated PLA_2_ secretion as revealed by immunoblotting analysis using sPLA_2_-IIE- and sPLA_2_-V-reacting antibodies. It is known that aged thioglycollate broth produces abundant advanced glycation end products (AGEs) via non-enzymatic reactions between proteins and reducing sugars. However, it is not yet known whether these AGEs similarly stimulate peritoneal macrophages to express sPLA_2_-IIE or sPLA_2_-V through RAGE, a receptor shared by SAA [12] and other proinflammatory ligands (such as HMGB1). In light of SAA’s capacity in upregulating Lp-PLA_2_ (sPLA_2_-VII) in human THP-1 monocytes [24] and sPLA_2_-IIA in smooth muscle cells [23], it now appears that SAA may participate in the regulation of distinct sPLA_2_s in different types of cells.

Consistent with HDL’s capacity in capturing SAA [27] and blocking its chemokine activities [20], we found that HDL also dose-dependently attenuated SAA-induced sPLA_2_-IIE/V expression, HMGB1 release and NO production. These findings further support the possibility that sPLA_2_ may potentiate the release of other late mediators including HMGB1 [7] and NO [6]. Notably, the SAA-induced NO production was entirely dependent on functional TLR4 signaling [15,29], because it was almost completely abolished in TLR4-deficient macrophages. In agreement with the notion that NO serves as a signaling molecule for autophagy induction [38], we found for the first time, that SAA effectively induced LC3-II production and aggregation—two markers of autophagy. Similarly, this SAA-induced LC3-II production and aggregation was attenuated by HDL, particularly when given at relative high concentrations (100 μg/ml), suggesting that HDL similarly counter-regulated SAA-induced autophagy.

In addition, HDL also prevented SAA-induced secretion of several cytokines (e.g., IL-6) and chemokines (e.g., MCP-1 and RANTES) in both RAW 264.7 cells and primary macrophages, confirming that RAW 264.7 cells and primary peritoneal macrophages do share similar cytokine responses. Interestingly, these HDL-inhibitable cytokines/chemokines may be similarly dependent on functional TLR4 signaling, since TLR4 disruption resulted in a marked reduction of SAA-induced secretion of IL-6, MCP-1 and RANTES [29]. In a sharp contrast, HDL did not markedly inhibit SAA-induced secretion of LIX, MIP-2 or G-CSF, which might depend on other receptor-dependent signaling pathways. Indeed, it has been shown that SAA induces G-CSF production via TLR2 activation in both primary macrophages and RAW 264.7 cells [14]. It is possible that HDL exerts its inhibitory effects via binding to the N-terminal α-helical domain (amino acid 1–28) of SAA, thereby possibly preventing its engagement with TLR4, but not TLR2 or other cell surface receptors. Because SAA may utilize distinct receptors to induce different cytokines, it may be possible to use HDL to impair distinct receptor pathways to counter-regulate specific inflammatory mediators.

At present, HDL and Apo-AI mimetic peptide have been proven protective against experimental sepsis [46–48] or human endotoxemia [49], and should be tested for patients with other inflammatory diseases [50]. Despite its remarkable anti-inflammatory properties, circulating HDL levels are often reduced in septic patients, and the magnitude of this reduction is positively correlated with the severity of the illness [51]. It is thus important to elucidate how HDL counter-regulates SAA and other proinflammatory mediators in order to provide guidance to future development of novel therapeutic strategies for inflammatory diseases.

In summary, we provided the first evidence that human SAA dramatically upregulates the expression and secretion of sPLA_2_-IIE and sPLA_2_-V in murine macrophage cultures. Furthermore, HDL dose-dependently attenuated SAA-induced secretion of sPLA_2_-IIE, sPLA_2_-V, HMGB1 and NO, reinforcing the notion that sPLA_2_ may potentiate the production of various late proinflammatory mediators. In addition, HDL attenuated SAA-induced secretion of a few TLR4-dependent cytokines (e.g., IL-6) and chemokines (e.g., MCP-1 and RANTES). It now appears that HDL counter-regulates SAA action by impairing its engagement with various receptors with different efficiencies. It is thus important to continue to elucidate how HDL counter-regulates SAA and other proinflammatory mediators in order to develop novel therapeutic strategy for various inflammatory diseases.

## Abbreviations

HDL: high-density lipoproteins
HMGB1: high mobility group box 1
LPS: lipopolysaccharide
NO: nitric oxide
sPLA_2_: secretory phospholipase A_2_
SAA: serum amyloid A
RAGE: receptor for advanced glycation end products
RT-PCR: reverse transcription polymerase chain reaction
TLR: toll-like receptors
